# Uptake, Willingness, and Determinants of Herpes Zoster Vaccination in Adults with Chronic Diseases in Riyadh, Saudi Arabia

**DOI:** 10.3390/healthcare13192495

**Published:** 2025-10-01

**Authors:** Haytham I. AlSaif, Lara AlMuaawi, Shaikhah Alsenani, Nouf Aldalaqan, Yara Mulia, Farah Alqazlan, Sara Alsheikh, Muath Alsaidan, Norah A. Alshehri, Abdullah A. Alrasheed

**Affiliations:** Department of Family and Community Medicine, College of Medicine, King Saud University, P.O. Box 2925, Riyadh 11461, Saudi Arabia; laraalmuaawi@gmail.com (L.A.); shaikhah5alsenan@gmail.com (S.A.); noufaldalaqan@gmail.com (N.A.); yaramul.2002@gmail.com (Y.M.); farah.alqazlan21@gmail.com (F.A.); saraalsheikh.19@gmail.com (S.A.); malsaidan@ksu.edu.sa (M.A.); drnora@ksu.edu.sa (N.A.A.); aalrasheed1@ksu.edu.sa (A.A.A.)

**Keywords:** herpes zoster, herpes zoster vaccine, vaccination coverage, prevention, healthcare acceptability, chronic disease, health knowledge, attitudes, practice, Saudi Arabia

## Abstract

**Background/Objectives**: Herpes zoster (HZ) poses a substantial risk to adults aged ≥ 50, particularly those with chronic disease, and may lead to postherpetic neuralgia. Following Saudi Arabia’s introduction of the recombinant zoster vaccine (RZV), we assessed the RZV’s uptake among adults ≥ 50 and their willingness to receive it and examined how knowledge and attitudes influenced these outcomes. **Methods**: In 2024, we conducted a cross-sectional, interview-based study in the outpatient clinics of a Riyadh tertiary hospital using a structured questionnaire adapted from the literature to assess knowledge, attitudes, and practices regarding HZ and RZV. **Results**: Among 333 participants, HZ vaccine uptake was low (12%). Among the unvaccinated, 45.7% (134/293) were willing to be vaccinated, and knowledge of HZ and RZV was low (mean: 3.84/14). Uptake was most strongly associated with physician recommendation (OR: 7.5; 95% CI: 2.79–20.11), followed by greater knowledge (OR: 1.42; 95% CI: 1.19–1.67). Willingness was best predicted by higher attitude scores (OR: 1.28; 95% CI: 1.12–1.48). The most reported barrier was low perceived risk (27%; n = 79). **Conclusions**: Uptake among adults aged ≥50 with chronic disease was low due to poor knowledge and low perceived risk, yet many were willing to be vaccinated. Enhancing physician–patient counseling and targeted campaigns addressing HZ risk and RZV misconceptions could increase uptake.

## 1. Introduction

The varicella zoster virus, the etiological agent of chickenpox (varicella), is usually contracted during childhood and remains latent in the neural ganglia. However, it can reactivate and manifest as herpes zoster (HZ) [1,2]. HZ generally presents as a localized, painful cutaneous eruption that can be complicated by postherpetic neuralgia, which may last from three months to several years [1]. In the general population, HZ incidence ranges from 3 to 5 per 1000 person-years and is comparable in North America, Europe, and the Asia–Pacific [3]. Based on data from the Saudi Ministry of Health (MOH), the annual HZ incidence was 13.1 cases per 100,000 patients in 2018 [4]. HZ incidence increases with age and in certain comorbidities, including diabetes mellitus, autoimmune diseases (e.g., systemic lupus erythematosus and rheumatoid arthritis), asthma, chronic obstructive pulmonary disease, hematologic and solid malignancies, and human immunodeficiency virus, as well as in hematopoietic cell and solid organ transplant recipients [3].

Vaccines effectively prevent HZ and its complications, such as postherpetic neuralgia [5]. The recombinant zoster vaccine (RZV) is recommended for immunocompromised individuals aged ≥19 years and immunocompetent individuals aged ≥50 years [6]. Although RZV is contraindicated for those with an allergy to its components, it exhibits major interactions only with rarely used treatments like immunotherapy and monoclonal antibodies [7,8]. Common RZV side effects include pain, myalgia, and fatigue [9]. A phase three trial found that the rates of serious adverse events were similar in the vaccinated versus placebo groups [10].

Between 2017 and 2019, 7,097,441 first RZV doses and 4,277,636 second RZV doses were administered in the United States (U.S.) [11]. In the Middle East, a survey conducted in the United Arab Emirates (U.A.E.) showed that 3.3% of participants received the RZV [12]. In Saudi Arabia, the MOH started offering the RZV to individuals aged ≥50 years in September 2022 [13]. Multiple self-administered surveys conducted in Western and Eastern Saudi Arabia, as well as nationally, reported RZV uptake ranging from 5.4% to 8% (administered online and in public settings) [14,15,16]. An online self-administered survey involving participants with diabetes in Qassim region, Saudi Arabia, found that 25.4% were willing to be vaccinated, with males showing more willingness [17].

Factors such as recommendation by a physician or healthcare practitioner can increase participants’ willingness to receive the HZ vaccine [12,14,17,18]. However, multiple barriers, including skepticism about a vaccine’s efficacy and safety, financial concerns, and the lack of awareness about the vaccine’s availability, prevent individuals from receiving HZ immunization [19]. Some groups, such as older people or those with lower educational or income levels, are likely to be less willing to receive HZ vaccination [19].

People aged ≥50 years, as well as those with chronic diseases, face a higher risk of HZ disease and its complications. However, previous studies in Saudi Arabia relied mostly on online self-administered surveys, which may not capture the views of older adults or those with low literacy. No hospital-based study has yet examined how knowledge and attitudes influence real-world RZV uptake among high-risk patients. Here, we aimed to measure the knowledge, attitudes, practices (especially uptake), and barriers surrounding HZ vaccination in the aforementioned population. We also examined how knowledge and attitudes towards HZ and its vaccine impact RZV uptake and people’s willingness to receive it. This study’s findings will improve our understanding of real-world uptake, which will guide healthcare provision for optimized protection from HZ and its complications in high-risk populations.

## 2. Materials and Methods

### 2.1. Setting and Participants

This cross-sectional study was conducted at King Khalid University Hospital (KKUH), an academic tertiary hospital in Northwestern Riyadh, the largest Saudi Arabian city. KKUH provides multidisciplinary primary, secondary, and tertiary care and has an outpatient building where patients can access various specialties, including general medicine (e.g., family medicine and internal medicine) and specialized clinics (e.g., diabetes and cardiology). This building functioned as the site for data collection, which was performed in March 2024. The inclusion criteria covered (i) registered KKUH patients (with an active medical record) presenting for a scheduled or walk-in outpatient appointment during the study period; (ii) age ≥50 years; and (iii) the ability to provide informed consent. The exclusion criteria ruled out (i) individuals < 50 years; (ii) non-patients (companions/visitors) and hospital staff; (iii) inpatients or emergency department attendees; and (iv) individuals unable to provide consent.

### 2.2. Study Instrument

A closed-ended structured questionnaire (in Arabic) was adapted from a similar United Arab Emirates study [12]. Four faculty members of the Department of Family and Community Medicine (H.I.A., M.A., N.A.A., and A.A.A.) assessed the questionnaire for content validity, including clarity, comprehensiveness, and item inclusion. A pilot study involving 25 participants was conducted to ensure comprehensibility and clarity. The domain assessing attitudes showed acceptable internal consistency (Cronbach’s alpha: 0.701). The questionnaire was modified and questions reordered based on feedback. The final version of the questionnaire featured the following domains with 28 questions: demographics (four questions), baseline clinical variables (five questions), knowledge of HZ and its vaccine (ten questions), participants’ knowledge sources (one question), attitudes towards HZ prevention (three questions rated on a Likert scale with one, two, and three indicating disagree, neutral, and agree, respectively), and practices surrounding the HZ vaccine and barriers to its use (five questions).

### 2.3. Data Collection

The sample size was calculated using the formula Z^2^ P(1 − P)/d^2^. The hypothesized proportion of participants who had received at least one dose of the HZ vaccine was 8% [19]; the targeted confidence interval was 95% and the margin of error was 3%, resulting in a target sample size of 314. Participants were selected in the waiting areas of the KKUH outpatient building through systematic random sampling by selecting only those on even-numbered seats and performing face-to-face interviews with those individuals. Six data collectors were trained by an associate professor (H.I.A.), and iterations were performed to decrease variability in interview and response recording. Data collectors obtained informed consent, read the questions aloud without additional interpretation, and allowed the participant to answer based on the given choices before directly entering participant responses into tablet devices using Google Forms (Google LLC, Mountain View, CA, USA).

### 2.4. Data Analyses

Knowledge on HZ and its vaccine was assessed using ten questions; eight of these each scored one point, and the remaining two (covering vaccine eligibility for three demographic groups and three methods of booking a vaccine) were scored a point per group or method, for a maximum knowledge score of 14 points. We categorized the scores similarly to the U.A.E. study: high (≥80%), intermediate (≥60%), moderate (≥40%), low (≥20%), and unsatisfactory (<20%) [12]. Attitude towards HZ prevention was covered by three questions (total score: 9), with higher attitude scores indicating higher perceived HZ infection risk and interest in knowing more about HZ and its prevention. Categorical variables were presented as frequencies and percentages, while quantitative variables were presented as means and interquartile ranges. In bivariate analysis, Pearson’s chi-square test was used for categorical variables and Student’s *t*-test was used for interval variables to compare the statistical significance of differences in the proportions or means between groups. Significant variables in the bivariate analysis were entered in a binomial logistic regression model for adjusted analysis. Categorical variables with response options “Yes,” “No,” and “I don’t know” were dichotomized as “Yes” and “No/Don’t know”. *p* < 0.05 indicated statistically significant results. Data were analyzed using SPSS version 25.0 (IBM Inc., Chicago, IL, USA).

### 2.5. Ethical Considerations

Permission to use the questionnaire was obtained from the authors of the U.A.E. study [12]. Permission to conduct the study at KKUH was granted by the Institutional Review Board of the College of Medicine at King Saud University on 15 January 2024 (Ref. No. 24/1060/IRB). All study participants gave written informed consent, and no identifiable data, e.g., names, contact information, or medical record numbers, were collected. All collected data were treated with confidentiality and stored on a password-protected computer by the principal investigators.

## 3. Results

### 3.1. Participant Characteristics

**Figure 1:**
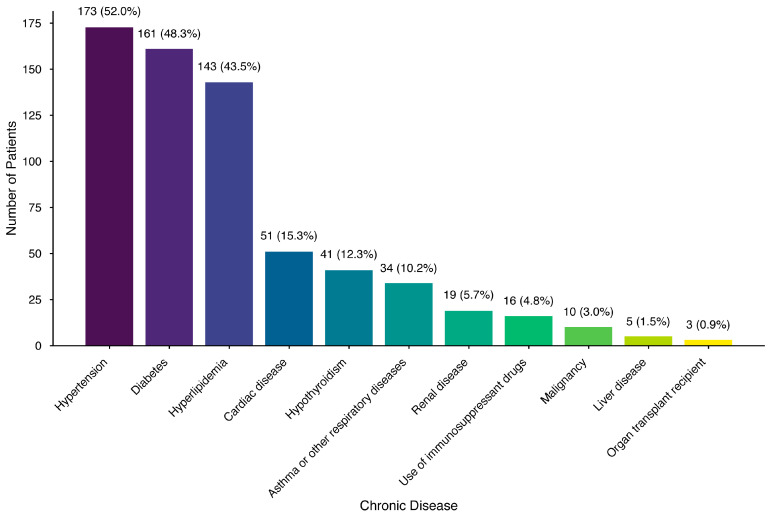
The frequency of the chronic diseases reported by the participants (n = 333).

Of the 350 participants we approached, 11 declined and 6 did not complete the interview; therefore, the analysis involved those 333 participants who completed the interview. The participants’ mean age was 60.4 ± 7.6 years (median: 60 years; range: 50–92 years). The highest education level was an undergraduate or postgraduate college degree (45.9% of participants). Of the participants, 46.5% had a history of chickenpox, 8.4% reported a history of HZ, and 30% had a family history of HZ. Most participants (83.5%) had at least one chronic disease. Table 1 describes the demographic and baseline clinical characteristics of the participants. The most prevalent reported chronic disease was hypertension (52%), followed by diabetes mellitus (48.3%). Figure 1 shows the frequency of the chronic diseases reported by the study participants.

### 3.2. The Knowledge of HZ and Its Vaccine

**Figure 2:**
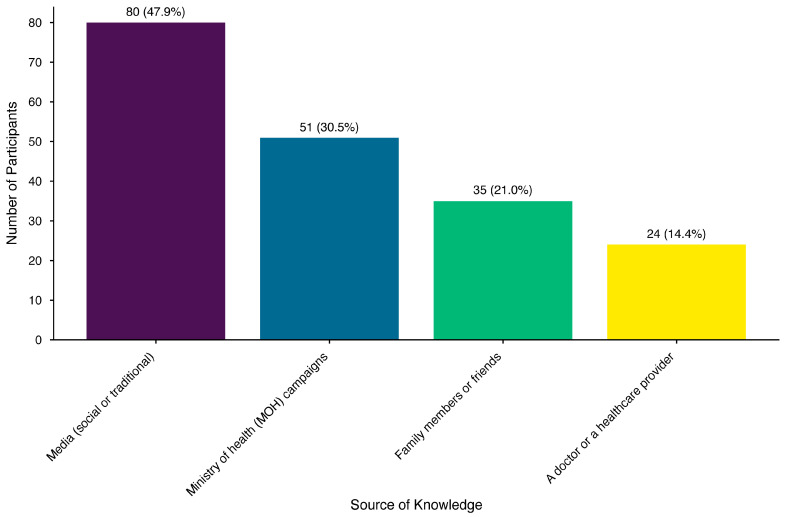
Participant-reported sources of knowledge about the herpes zoster vaccine (n = 333).

The mean knowledge score was 3.84 ± 3.35 points (median: 3; interquartile range: 1–7 points). The difference in mean knowledge scores was significant across age groups (F [2, 330] = 4.652, *p* = 0.01), with participants in the 56–60-year age group having the highest mean (4.47 ± 3.53). Only half of the participants (50.2%) knew that HZ can cause complications like postherpetic neuralgia. A third of the participants (30.3%) identified the appropriate age for HZ vaccination correctly. A small proportion of the participants (7.2%) correctly answered the question on the impact of a history of chickenpox or HZ infection on HZ vaccination eligibility. More than half of the participants (52.9%) were unaware that the Saudi MOH freely provides the HZ vaccine. Almost two-thirds of the participants (62.8%) did not know how to book an appointment for an HZ vaccination. The participants’ responses to the questions about knowledge on HZ and its vaccine are shown in Table 2. Traditional and social media (47.9%) were the most common sources of knowledge about HZ and its vaccine, and the least-cited source of knowledge was a doctor or healthcare provider (14.4%). Figure 2 shows the frequency of participant-reported sources of knowledge about the HZ vaccine.

### 3.3. Attitude Towards HZ Prevention

**Figure 3:**
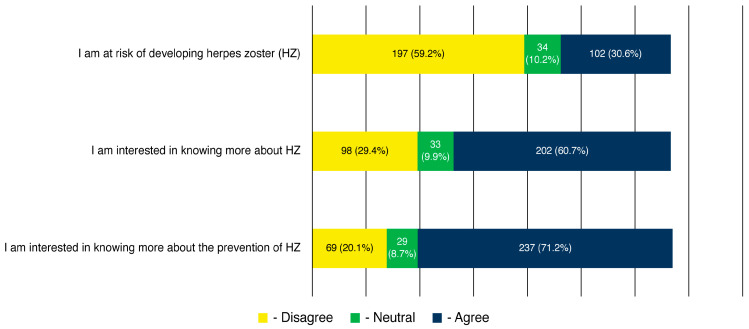
Participants’ attitude towards herpes zoster infection and its prevention (n = 333).

The mean attitude score was 6.54 ± 2.03 points (median: 7; interquartile range: 5–8). Although 59.2% of the participants did not consider themselves at risk of HZ, 60.7% were interested in obtaining more information about the disease, and 71.2% showed interest in learning more about the HZ vaccine. Figure 3 shows the participants’ responses regarding their attitudes towards HZ and its vaccine.

### 3.4. Practices Towards the HZ Vaccine

Slightly more participants received the HZ vaccine (12%) than were recommended it by their doctors (9.6%). Most participants (77.5%) received the HZ vaccine at the MOH primary care centers, and 52.5% completed the vaccine series with two doses. Among unvaccinated participants, 45.7% were willing to receive the HZ vaccine. More than half of the unvaccinated participants (51.2%) had no barriers to vaccination, while less than half of the participants (48.8%) had at least one barrier to HZ vaccination. A lack of perceived risk due to current health status was the most common barrier (27%) to receiving HZ vaccination. Table 3 shows the frequency of participants’ practices regarding HZ vaccination.

### 3.5. Predictors of HZ Vaccine Uptake

Bivariate analysis revealed that the following variables were significantly associated with vaccination against HZ: age group, educational attainment, presence of chronic diseases, doctor recommendation of HZ vaccination, knowledge score, and attitude score. A binomial logistic regression model was used to determine the effects of these variables on the likelihood of receiving HZ vaccination. Participants who received a doctor’s HZ vaccine recommendation were likelier to receive the vaccine than those who did not (odds ratio [OR]: 7.5; 95% confidence interval [CI]: 2.79–20.11). The likelihood of receiving the HZ vaccine increased 1.41 times with each one-point increase in the knowledge score (OR: 1.41; 95% CI: 1.19–1.67). Participants aged 56–60 years (OR: 4.56; 95% CI: 1.28–16.29) and ≥61 years (OR: 4.02; 95% CI: 1.16–13.94) had a higher likelihood of receiving the HZ vaccine than those aged 50–55 years. Participants who received influenza vaccination were 3.5 times more likely to receive the HZ vaccine than those who did not (OR: 3.5; 95% CI: 1.31–9.37). Table 4 shows the results of the binomial regression model of the variables associated with HZ vaccination uptake.

### 3.6. Predictors of the Willingness to Receive the HZ Vaccine

Bivariate analysis revealed a significant association between the willingness to receive HZ vaccination and the attitude score, knowledge score, influenza vaccination, family history of HZ, educational attainment, and personal history of chickenpox. Binomial logistic regression was used to assess the effects of these variables on the likelihood of being willing to receive the HZ vaccine. For each one-point increase in the attitude score, the likelihood of being willing to receive the HZ vaccine increased 1.28 times (OR: 1.28; 95% CI: 1.12–1.48). For each one-point increase in the knowledge score, the likelihood of being willing to receive the HZ vaccine increased 1.13 times (OR: 1.13; 95% CI: 1.03–1.24). Finally, participants who received influenza vaccination were 1.94 times more likely to be willing to receive the HZ vaccine than those who did not (OR: 1.94; 95% CI: 1.15–2.26). Table 5 shows the results of the binomial regression model analysis of the variables associated with the willingness to receive the HZ vaccine. Bivariate analyses results are presented in Appendix A (Table A1, Table A2 and Table A3).

## 4. Discussion

This interview-based design captured the opinions of participants with low literacy who would likely be missed by self-administered surveys. To our knowledge, this is the first estimate of HZ vaccine uptake within one year of RZV introduction in Riyadh, Saudi Arabia. Our sample had a high burden of chronic disease (83.3%), higher than Western Saudi Arabia (44.4%) and the U.A.E. (66.6%) [12,14]. Uptake among adults aged ≥50 was 12%, exceeding prior reports from Saudi Arabia (5.4–8%) and the U.A.E. (3.3%) [12,14,15,16], likely reflecting differences in recruitment/target populations [14,15,16] and the more recent timing of our study. Nevertheless, coverage in our sample remains lower than in the United States, where RZV uptake reached 18.6% among adults aged ≥50 years by 2021, four years after its approval [20]. In Japan, municipal subsidy programs in 2022 achieved 2.97% uptake—lower than our estimate—plausibly because subsidies did not eliminate copayments, whereas RZV is offered without copayment in Saudi Arabia [21]. Overall, cross-country differences appear driven primarily by program maturity and cost.

Uptake was highest in adults aged 56–60 years, mirroring a Saudi online survey [15]; this group also had the highest HZ and RZV knowledge, which may partly explain their greater uptake. Prior influenza vaccination was associated with higher RZV uptake and greater willingness in our sample and among patients attending a U.S. dermatology clinic [22], but not in a U.S. national survey or among patients attending infectious disease clinics in South Korea [23,24]. These discrepancies may reflect the influence of positive prior vaccination experiences and greater exposure to healthcare providers. Physician recommendation consistently predicts HZ vaccination [12,14,17,23,25,26,27,28], yet only 9.6% of participants reported receiving one. Future research should identify and address barriers that limit physicians’ vaccine counseling to increase uptake.

Willingness to receive the vaccine among the unvaccinated was 45.7%, below the global estimate of 55.74% (95% CI: 40.85–70.13%) [19]. This may reflect that some of the most willing individuals (12%) had already been vaccinated and were therefore excluded from the willingness estimate. The most cited barrier was low perceived risk; only 50.2% reported knowledge on HZ’s complications despite the participants’ high burden of chronic disease. By contrast, a Shanghai study reported greater willingness among those with underlying diseases [29]. Consistent with evidence linking willingness to perceived severity and susceptibility [19], physicians should emphasize HZ’s risks and complications, especially for patients with chronic conditions. Concerns about potential interactions between RZV and current medications were common, mirroring reports among patients with autoimmune rheumatologic disease regarding COVID-19 vaccination [30]. Given participants’ high burden of chronic disease and the immunosuppressive effects of some therapies, these concerns are noteworthy, as such treatments increase the risk of HZ and its complications.

Nearly half (49.8%) had never heard of the HZ vaccine—consistent with local estimates (44.2–46.6%) [14,17]—and 13.3% cited this lack of knowledge about the existence of the HZ vaccine as a barrier. Higher uptake depends on public awareness that the vaccine exists [31]. Over half (52.9%) were unaware that the MOH provides the RZV free of charge, which offsets the financial barrier. Furthermore, 62.8% did not know how to book a vaccination appointment, indicating substantial practical barriers. Although the attitude score was the strongest predictor of willingness, it did not predict uptake, likely reflecting unaddressed safety concerns (e.g., adverse effects or drug interactions), practical barriers (e.g., appointment booking), and the vaccine’s recent introduction.

Most vaccinations were administered at MOH primary healthcare centers. Expanding vaccination across different healthcare provision sectors is a system-level strategy to increase vaccination uptake [32]. The Ministry of Health and other healthcare sectors could expand vaccination through community pharmacies and community-based mobile clinics, an approach that systematic reviews have shown to increase uptake [33]. Only 52.5% completed the two-dose series, likely reflecting recency; patient-directed measures (education, scheduled follow-ups, reminders) improve completion [34]. In parallel, healthcare worker-directed interventions are also important. Tailored reminders and multicomponent strategies (combining two or more approaches) effectively support HCWs in addressing vaccines with older adults [35]. For example, interventions that integrated provider education with electronic reminders and audit feedback achieved greater improvements in vaccination uptake than single-component approaches, whereas education-only interventions were found to be less effective [35].

Findings can guide clinicians and public health officials to enhance HZ vaccine uptake. Recurring campaigns via social and traditional media emphasizing HZ risk and RZV safety are needed. Efforts should expand access beyond MOH primary care (e.g., additional provider sites) and encourage routine physician–patient discussions, especially for those with chronic diseases; preventive visits are key opportunities. Limitations include self-report (susceptible to recall and social desirability biases), single-center sampling (introducing selection bias and limiting generalizability), and a cross-sectional design (precluding causal inference). We did not measure potential confounders (e.g., socioeconomic status, access to care, or cultural attitudes). Our regression models showed moderate explanatory power. Vaccine decision-making is influenced by multifactorial and context-dependent elements that were not fully captured by our instrument. Further research should test interventions to increase physician–patient HZ vaccination discussions and reduce barriers to counseling, while incorporating broader psychosocial, cultural, and system-level factors to better explain residual variance in uptake and willingness.

## 5. Conclusions

This study revealed a low HZ vaccine uptake among participants aged ≥50 years who had a high burden of chronic diseases. However, less than half of the unvaccinated individuals expressed willingness to receive the vaccine, indicating the potential for increased coverage. Physician recommendation was the strongest predictor of uptake. Encounters with healthcare providers therefore represent critical opportunities for vaccination discussions. Addressing barriers that limit these discussions and implementing reminders and multicomponent strategies may substantially increase uptake. A low perceived risk of HZ and its complications is a major factor that needs to be addressed by healthcare providers and awareness campaigns. Expanding and facilitating access to vaccination might increase uptake, ultimately reducing HZ-related morbidity.

## Figures and Tables

**Table 1:** The demographic and clinical characteristics of the study participants (n = 333).

Characteristic		Frequency	Percentage
Sex	Male	175	52.6%
	Female	158	47.4%
Age group (years)	50–55	103	30.9%
	56–60	91	27.3%
	≥61	139	41.8%
Education level	Illiterate	50	15%
	School (elementary, middle, high)	130	39%
	College (undergraduate, postgraduate)	153	45.9%
Personal history of chickenpox	Yes	155	46.5%
	No	118	35.4%
	I do not know	60	18%
Ever heard about HZ	Yes	286	85.9%
	No	43	12.9%
	I do not know	4	1.2%
Personal history of HZ	Yes	28	8.4%
	No	259	77.8%
	I do not know	46	13.8%
History of HZ in a family member	Yes	100	30%
	No	184	55.3%
	I do not know	49	14.7%
Presence of chronic diseases	Yes	278	83.5%
	No	55	16.5%

HZ, herpes zoster.

**Table 2:** Participants’ knowledge about herpes zoster infection and its vaccine (n = 333).

Question		Frequency	Percentage
Lifetime risk of having HZ is up to one third	Yes	79	23.7%
	No	30	9%
	I do not know	224	67.3%
Immunocompromised individuals are at higher risk of HZ	Yes	186	55.9%
	No	8	2.4%
	I do not know	139	41.7%
HZ might cause complications such as postherpetic neuralgia	Yes	167	50.2%
	No	10	3%
	I do not know	156	46.8%
Ever heard about HZ vaccine	Yes	167	50.2%
	No	166	49.8%
The HZ vaccine reduces the incidence of HZ by more than 50%	True	107	32.1%
	False	3	0.9%
	I do not know	223	67%
HZ vaccine can treat active HZ	True	37	11.1%
	False	58	17.4%
	I do not know	238	71.5%
Age groups recommended for vaccination against HZ (in the absence of comorbidities)	All age groups	16	4.8%
	≥18 years	5	1.5%
	≥50 years	101	30.3%
	≥60 years	13	3.9%
	I do not know	198	59.5%
Which group/s can take the HZ vaccination? (more than one choice)	Did not have chickenpox	66	19.8%
	Had chickenpox	33	9.9%
	Had HZ	25	7.5%
	I do not know	244	73.3%
The Saudi MOH provides the HZ vaccine for free	True	157	47.1%
	I do not know	176	52.9%
Methods to book an appointment for the HZ vaccine through MOH (more than one choice)	MOH phone (937)	14	4.2%
	MOH mobile application (Sehaty)	69	20.7%
	In-person at the primary care center	49	14.7%
	I do not know	209	62.8%

HZ, herpes zoster, MOH, Ministry of Health.

**Table 3:** Participants’ practices towards herpes zoster vaccine and other vaccines (n = 333).

Question		Frequency	Percentage
Vaccines previously received (more than one choice)	COVID-19	321	96.4%
	Influenza	166	49.8%
	Hepatitis B	38	11.4%
	Pneumococcal	6	1.8%
	None	12	3.6%
Your doctor recommended the HZ vaccine to you	Yes	32	9.6%
	No	294	88.3%
	I do not know	7	2.1%
Vaccinated against HZ	Yes	40	12%
	No	289	86.8%
	I do not know	4	1.2%
Vaccinated participants (n = 40)			
Where were you vaccinated	MOH primary care center	31	77.5%
	Other public sector healthcare institutions	7	17.5%
	King Saud University Medica City	2	5%
	Private sector healthcare institution	0	0
Doses of the HZ vaccine received	one	19	47.5%
	two	21	52.5%
Unvaccinated participants (n = 293)			
Willing to get vaccinated against HZ	Yes	134	45.7%
	No	122	41.7%
	I do not know	37	12.6%
Barriers towards vaccination (more than one choice)	Not at risk because I am healthy	79	27%
	Concerned about the side effects of the vaccine	72	24.6%
	Would rather get treatment when I get sick	44	15%
	Did not know that the vaccine existed	39	13.3%
	Concerned about the interaction between the HZ vaccine and my medications	37	12.6%
	Do not believe in vaccines	23	7.8%
	Had an allergic reaction after receiving an injection	8	2.7%

HZ, herpes zoster, COVID-19, coronavirus disease of 2019.

**Table 4:** Binomial logistic regression analysis of the association between doctor recommendation, knowledge score, age group, influenza vaccination, chronic diseases, educational attainment, attitude score, and uptake of the herpes zoster vaccine *.

	B	S.E.	Wald	*p*-Value	OR	95% CI for OR	
						Lower	Upper
Constant	−8.665	1.829	22.445	<0.001	0		
Doctor recommended receiving the HZ vaccine (ref. no/I do not know)	2.014	0.504	15.995	<0.001	7.495	2.793	20.111
Knowledge score (ref. 0)	0.344	0.086	15.852	<0.001	1.41	1.191	1.67
Age 56–60 years (ref. 50–55 years)	1.517	0.65	5.456	0.019	4.559	1.277	16.285
Age ≥ 61 years (ref. 50–55 years)	1.39	0.635	4.794	0.029	4.016	1.157	13.94
Previously received influenza vaccine (ref. no vaccine)	1.252	0.502	6.217	0.013	3.499	1.307	9.365
Presence of chronic diseases (ref. no)	1.855	1.13	2.692	0.101	6.39	0.697	58.568
School degree (ref. illiterate)	0.487	1.12	0.189	0.663	1.628	0.181	14.61
College degree (ref. illiterate)	1.022	1.097	0.869	0.351	2.779	0.324	23.84
Attitude score (ref. 3)	−0.004	0.128	0.001	0.974	0.996	0.775	1.28

B: unstandardized regression coefficient, S.E.: standard error, OR: odds ratio, CI: confidence interval. * Nagelkerke R^2^ = 0. 0.473.

**Table 5:** Binomial logistic regression for the association between the willingness to receive the herpes zoster vaccine * and attitude score, knowledge score, influenza vaccination, family history of herpes zoster, educational attainment, and a personal history of chickenpox.

	B	S.E.	Wald	*p*-Value	OR	95% CI for OR	
						Lower	Upper
Constant	−4.187	0.8541	24.029	<0.001	0.0152		
Attitude Score (ref. 3)	0.248	0.0715	12.0325	<0.001	1.2815	1.1165	1.479
Knowledge score (ref. 0)	0.1218	0.0458	7.068	0.008	1.1296	1.0333	1.2373
Previously received influenza vaccine (ref. no vaccine)	0.6609	0.2649	6.2269	0.013	1.9366	1.1548	3.2684
History of herpes zoster in a family member (ref. no/I do not know)	0.5921	0.309	3.6723	0.055	1.8078	0.9876	3.3282
School degree (ref. illiterate)	0.4496	0.396	1.2889	0.256	1.5676	0.73	3.4754
College degree (ref. illiterate)	0.5096	0.3963	1.6534	0.199	1.6647	0.773	3.6855
Personal history of chickenpox (ref. no/I do not know)	0.4471	0.268	2.7824	0.095	1.5637	0.9257	2.6531

B: unstandardized regression coefficient, S.E.: standard error, OR: odds ratio, CI: confidence interval. * Nagelkerke R^2^ = 0.255.

## Data Availability

All original contributions from this study are contained within the article; additional information is available from the corresponding author upon request.

## Abbreviations

The following abbreviations are used in this manuscript:

HZ	Herpes zoster
RZV	Recombinant zoster vaccine
MOH	Ministry of Health
KKUH	King Khalid University Hospital
OR	Odds ratio
CI	Confidence interval
U.S.	United States
U.A.E.	United Arab Emirates
SPSS	Statistical Package for the Social Sciences
COVID-19	Coronavirus disease of 2019
IRB	Institutional Review Board

## Appendix A. Bivariate Analyses of the Associations Between Demographic and Clinical Variables and the Uptake of the Herpes Zoster Vaccine, Willingness to Receive It, and Knowledge and Attitude Scores

**Table A1:** Bivariate analysis of the association between demographic and clinical variables and herpes zoster vaccination (n = 333).

			Vaccinated Against Herpes Zoster (HZ)			
Variable			Yes	No/I Do Not Know	Total	*p*-Value
Sex	Male	Frequency (%)	24 (13.7%)	151 (86.3%)	175	0.315
	Female		16 (10.1%)	142 (89.9%)	158	
Age group (years)	50–55		5 (4.9%)	98 (95.1%)	103	0.018
	56–60		16 (17.6%)	75 (82.4%)	91	
	More than 60		19 (13.7%)	120 (86.3%)	139	
Education level	Illiterate		1 (2%)	49 (98%)	50	0.006
	School degree		12 (9.2%)	118 (90.8%)	130	
	College degree		27 (17.6%)	126 (82.4%)	153	
Presence of chronic diseases	Yes		39 (14%)	239 (86%)	278	0.011
	No		1 (1.8%)	54 (98.2%)	55	
Nationality	Saudi		37 (12.3%)	264 (87.7%)	301	0.629
	Non-Saudi		3 (9.4%)	29 (90.6%)	32	
Personal history of chickenpox	Yes		21 (13.5%)	134 (86.5%)	155	0.421
	No/I do not know		19 (10.7%)	159 (89.3%)	178	
Personal history of herpes zoster (HZ)	Yes		3 (10.7%)	25 (89.3%)	28	0.825
	No/I do not know		37 (12.1%)	268 (87.9%)	305	
History of HZ in a family member	Yes		16 (16%)	84 (84%)	100	0.143
	No/I do not know		24 (10.3%)	209 (89.7%)	233	
Your doctor recommended the HZ vaccine to you	Yes		16 (50%)	16 (50%)	32 (100%)	<0.001
	No/I do not know		24 (8%)	277 (92%)	301 (100%)	
Mean knowledge score			7.35 ± 1.88	3.36 ± 3.22		<0.001
Mean attitude score			7.2 ± 1.9	6.45 ± 2.04		0.028

**Table A2:** Bivariate analysis of the association between demographic and clinical variables and the willingness to be vaccinated against HZ (n = 333).

			Willing to Get Vaccinated Against Herpes Zoster (HZ)			
Variable			Yes	No/I Do Not Know	Total	*p*-Value
Age group (years)	50–55	Frequency (%)	45 (45.9%)	53 (54.1%)	98	0.633
	56–60		31 (41.3%)	44 (58.7%)	75	
	≥60		58 (48.3%)	62 (51.7%)	139	
Sex	Male		76 (50.3%)	75 (49.7%)	151	0.103
	Female		58 (40.8%)	84 (59.2%)	142	
Education level	Illiterate		14 (28.6%)	35 (71.4%)	49	0.009
	School degree		52 (44.1%)	66 (55.9%)	118	
	College degree		68 (54%)	58 (46%)	126	
Presence of chronic diseases	Yes		110 (46%)	129 (54%)	239	0.833
	No		24 (44.4%)	30 (55.6%)	54	
Nationality	Saudi		121 (45.8%)	143 (54.2%)	264	0.918
	Non-Saudi		13 (44.8%)	16 (55.2%)	29	
Personal history of chickenpox	Yes		71 (53%)	63 (47%)	134	0.022
	No/I do not know		63 (39.6%)	96 (60.4%)	159	
Personal history of herpes zoster (HZ)	Yes		12 (48%)	13 (52%)	25	0.812
	No/I do not know		122 (45.5%)	146 (54.5%)	268	
History of HZ in a family member	Yes		54 (64.3%)	30 (35.7%)	84	<0.001
	No/I do not know		80 (38.3%)	129 (61.7%)	209	
Your doctor recommended the HZ vaccine to you	Yes		11 (68.7%)	5 (31.3%)	16	0.057
	No/I do not know		123 (44.4%)	154 (55.6%)	277	
Mean knowledge score			4.45 ± 3.24	2.44 ± 2.91		<0.001
Mean attitude score			7.16 ± 1.71	5.85 ± 2.1		<0.001

**Table A3:** Mean knowledge and attitude scores across demographic and clinical variables (n = 333).

Variable		Mean Knowledge Score	*p*-Value *	Mean Attitude Score	*p*-Value *
Age group (years)	50–55	4.14 ± 3.28	0.01	6.87 ± 1.94	0.045
	56–60	4.47 ± 3.53		6.63 ± 1.98	
	≥60	3.2 ± 3.19		6.23 ± 2.1	
Sex	Male	3.87 ± 3.43	0.835	6.52 ± 2.05	0.869
	Female	3.8 ± 3.27		6.56 ± 2.02	
Education level	Illiterate	2 ± 2.75	<0.001	5.9 ± 2.04	0.001
	School degree	3.36 ± 3.28		6.3 ± 2.11	
	College degree	4.84 ± 3.26		5.95 ± 1.88	
Presence of chronic diseases	Yes	3.99 ± 3.37	0.058	6.58 ± 2.05	0.402
	No	3.05 ± 3.16		6.33 ± 1.93	
Nationality	Saudi	3.93 ± 3.39	0.097	6.5 ± 2.02	0.371
	Non-Saudi	3 ± 2.89		6.84 ± 2.19	
Personal history of chickenpox	Yes	3.98 ± 3.36	0.469	6.81 ± 1.95	0.021
	No/I do not know	3.71 ± 3.34		6.3 ± 2.08	
Personal history of herpes zoster (HZ)	Yes	5.21 ± 3.38	0.023	6.21 ± 2.06	0.38
	No/I do not know	3.71 ± 3.32		6.57 ± 2.03	
History of HZ in a family member	Yes	5.47 ± 2.92	<0.001	6.91 ± 2.07	0.028
	No/I do not know	3.14 ± 3.28		6.38 ± 2	
Your doctor recommended the HZ vaccine to you	Yes	7 ± 2.24	<0.001	6.94 ± 2.06	0.242
	No/I do not know	3.5 ± 3.27		6.5 ± 2.03	

* *t* test or one-way analysis of variance (ANOVA).
